# Mitigating Heat Stress in Broilers: Effects of *Bacillus subtilis* Probiotic and Garlic (*Allium sativum*) Supplementation on Growth Performance, Antioxidant Status, Cecal Microbiota and Immune Response

**DOI:** 10.1016/j.psj.2025.105795

**Published:** 2025-09-03

**Authors:** Hanan Al-Khalaifah, Muhammad Mushtaq, Muhammad Shoib, Imran Ullah, Muqadar Shah, Shabana Naz, Rifat Ullah Khan, Ala Abudabos, Ibrahim A. Alhidary

**Affiliations:** a Environment and Life Sciences Research Centre, Kuwait Institute for Scientific Research, Safat, Kuwait; b Department of Poultry Science, Faculty of Animal Husbandry and Veterinary Sciences, The University of Agriculture, Peshawar, Pakistan; c College of Veterinary Sciences, Faculty of Animal Husbandry and Veterinary Sciences, The University of Agriculture, Peshawar, Pakistan; d Department of Zoology, Government College, University, Faisalabad, Pakistan; e Department of Food and Animal Sciences, College of Agriculture, Tennessee State University, Nashville, TN 37209, USA; f Department of Animal Production, College of Food and Agriculture Science, King Saud University, Riyadh, Saudi Arabia

**Keywords:** Broilers, Bacillus subtilis, Garlic, Heat stress, Growth performance

## Abstract

This study evaluated the effects of *Bacillus subtilis* and garlic supplementation on growth performance, oxidative stress, and immune response in heat-stressed broilers. A total of 320-day-old Hubbard broiler chicks were randomly allocated to four dietary treatments: a control group (thermoneutral) and three heat-stressed groups supplemented with *Bacillus subtilis* at 3 × 10¹⁰, 4 × 10¹⁰, and 5 × 10¹⁰ CFU/kg each combined with 1 % garlic powder. Heat stress was induced by maintaining the temperature at 32–35°C for three hours daily from day 22-35. Broilers were evaluated for feed intake (FI), weight gain (WG), feed conversion ratio (FCR), malondialdehyde (MDA) levels, cecal microbiota composition, and differential leukocyte counts (DLC). The results showed that higher levels of *Bacillus subtilis* (4 × 10¹⁰ and 5 × 10¹⁰ CFU/kg) with 1 % garlic significantly (*P* < 0.001) improved FI, WG, and FCR during the finisher phase. Supplemented groups exhibited lower MDA levels, indicating reduced oxidative stress (*P* < 0.001). Moreover, DLC analysis revealed enhanced immune function, with reduced heterophil counts and increased monocyte and eosinophil percentages (*P* < 0.05). No significant differences were observed in cecal microbiota composition. Overall, 5 × 10¹⁰ CFU/kg *Bacillus subtilis* + 1 % garlic showed the best performance, demonstrating its potential as an effective strategy to mitigate heat stress in broilers.

## Introduction

Heat stress is a major challenge in poultry production, particularly in regions with high ambient temperatures, as it negatively affects feed intake, growth performance, immune function, and overall health (Khan et al., 2023; Al-Suwailem et al., 2024). Environmental stressors, such as excessive heat, can lead to oxidative stress, compromising cellular functions and increasing susceptibility to disease (Hafeez et al., 2021; Fatima et al., 2022; Hassan et al., 2022). Consequently, nutritional interventions, including probiotics and phytogenic feed additives, have been explored to mitigate the detrimental effects of heat stress on broilers (Ajmal et al., 2023; Magnoli et al., 2024; Fathanah et al., 2024; Khan et al., 2025).

*Bacillus subtilis* is a spore-forming probiotic that enhances intestinal health by modulating the gut microbiota, improving nutrient digestibility, and strengthening immune responses (Usman et al., 2024). It has been reported to increase *Lactobacillus spp.* while suppressing pathogenic bacteria such as *Escherichia coli* and *Salmonella*, thereby improving intestinal barrier integrity (Glago et al., 2024; Nguyen et al., 2024). Additionally, probiotics contribute to enzyme secretion, bile salt metabolism, and competitive exclusion of pathogens, making them effective alternatives to antibiotic growth promoters (Rashid et al., 2023; Gul and Alsayeqh, 2023; Phupaboon et al., 2024; Susalam et al., 2024; Yu et al., 2024a).

Garlic (*Allium sativum*) is widely recognized for its antimicrobial, antioxidant, and immunomodulatory properties (Liang-liang et al., 2023). The bioactive compounds in garlic, such as allicin, diallyl sulfides, and flavonoids, have been shown to improve feed efficiency and weight gain in broilers under stress conditions (Çalışkan and Emin, 2023). Furthermore, garlic supplementation reduces malondialdehyde (MDA) levels, an indicator of lipid peroxidation, thereby enhancing oxidative stability in broiler tissues (Khan et al., 2012a).

Several studies have demonstrated the individual effects of probiotics and garlic on broiler performance, but limited research exists on their combined effects, particularly under heat stress conditions. Probiotics and phytogenics may exert synergistic effects, where probiotics enhance gut microbial balance, and garlic provides additional antioxidant and immune-boosting benefits (Hafeez et al., 2024; Yu et al., 2024b; Basit et al., 2025). **For instance**, Hafeez et al. (2024) **reported improved growth and immune status with Bacillus subtilis supplementation alone, while** Yu et al. (2024b) **found that garlic powder enhanced antioxidant capacity and reduced lipid peroxidation in heat-stressed broilers. Similarly**, Basit et al. (2025) **demonstrated that probiotics and phytogenics improved gut health and nutrient digestibility, but their trial did not focus on combined supplementation or economic outcomes.** However, the optimal dosages and interactions between these additives require further investigation to maximize poultry performance. **Unlike these previous studies, the present trial specifically evaluates the synergistic potential of Bacillus subtilis combined with garlic under heat stress, while also integrating a broader set of response criteria including growth performance (feed intake, weight gain, FCR), oxidative stress markers (MDA levels), differential leukocyte count, cecal microbiota, and economic profitability. This provides a more comprehensive assessment of their combined efficacy compared to earlier studies.**

## Materials and methods

### Experimental Design and Management Practices

A total of 320-day-old broiler chicks (Hubbard) were randomly assigned to four treatment groups, with each group consisting of five replications of 16 chicks. The birds were housed in an open-sided poultry facility, ensuring adequate ventilation. During the brooding phase, all chicks were provided with a temperature-controlled environment using an incandescent lamp as a heat source. The brooding temperature was maintained at 32–35°C during the initial days and gradually reduced to 22 to 24°C. All birds were housed in the same facility but in separate pens under similar management conditions. On day 22, except for the control group that remained under thermoneutral conditions (24 ± 2°C), all treatment groups were exposed to heat stress by raising the room temperature to 32–35°C (average 34°C) for three hours daily using additional infrared heat lamps.

The birds were divided into the following four dietary treatments**:**

• Group A (Control)**:** Standard basal diet without supplementation (Table 1)

**Table 1:** Ingredients and chemical composition of basal feed.

Ingredients	Starter Phase (1-21 days)	Finisher phase (22-42 days)
Yellow corn	53.21	60.75
Soybean meal (48 % CP)	37.92	25.00
Corn gluten meal (34 % CP)	2.00	7.10
Corn oil	2.20	2.80
Dicalcium phosphate	2.30	2.05
Limestone	0.83	0.68
NaCl	0.45	0.50
Vitamin and minerals premix^1^	0.50	0.50
DL-Methionine	0.20	0.10
L-Lysine HCl	0.22	0.37
L-Threonine	0.11	0.10
Choline chloride	0.05	0.05
Chemical composition		
ME, kcal/kg	3000	3150
Crude protein, %	22.5	21.30
Methionine, %	0.55	0.44
Lysine, %	1.42	1.23
Sulfur amino acids, %	0.96	0.80
Threonine, %	0.95	0.85
Calcium, %	1.05	0.90
Available phosphorus, %	0.50	0.45

1 Provided per kg of diet: vitamain A, 8000 IU; vitamain D3,2000 IU; vitamain E,15 IU; vitamain K3, 1.5 mg; thiamine, 2 mg; riboflavin, 5 mg; pyridoxine, 5 mg; vitamain B12, 0.02 mg; folic acid, 0.7 mg; nicotinic acid, 40 mg; pantothenic acid, 12 mg; biotin, 0.2 mg b Provided per kg of diet: copper, 10 mg (as copper sulfate); iron, 90 mg (as ferrous sulfate); manganese, 100 mg (as manganese sulfate); zinc, 100 mg (as zinc sulfate); selenium, 0.3 mg (as sodium selenite); iodine, 0.5 mg (as calcium iodate).
• Group B: Diet supplemented with Bacillus subtilis at 3 × 10¹⁰ CFU/kg **+** 1 % garlic dry powder**.**
• Group C: Diet supplemented with Bacillus subtilis at 4 × 10¹⁰ CFU/kg **+** 1 % garlic dry powder**.**
• Group D: Diet supplemented with Bacillus subtilis at 5 × 10¹⁰ CFU/kg **+** 1 % garlic dry powder**.**

Bacillus subtilis was obtained from CLOSTAT® (Kemin Industries, Inc., Des Moines, IA, USA). **All experimental diets were prepared by thoroughly mixing the additives with the basal diet using a horizontal feed mixer to ensure uniform distribution. Each treatment diet was manufactured separately rather than top-dressed, and the required amounts of garlic powder and Bacillus subtilis were incorporated into the premix before final mixing.** The experiment lasted six weeks. Birds were maintained on a continuous lighting schedule for the first 24 hours, followed by 23 hours of light and 1 hour of darkness per day throughout the study. A standard commercial broiler diet was provided *ad libitum*, ensuring optimal nutrient availability. Fresh, clean drinking water was available ad libitum throughout the experiment, supplied via an automatic watering system. Standard management practices were followed to ensure the well-being and optimal performance of the birds under experimental conditions.

### Zootechnical Parameters

Daily feed consumption was recorded by measuring the total amount of feed offered and subtracting the leftover feed at the end of each day. The overall feed intake was calculated at the end of the trial. Daily water consumption was monitored by measuring the total amount of water provided and subtracting the remaining water at the end of the day. The cumulative water intake was determined at the conclusion of the study. The birds were weighed weekly to assess body weight gain. This was determined by subtracting the initial body weight from the final body weight at the end of each week. The feed conversion ratio was calculated weekly for each treatment group by dividing the total amount of feed consumed by the total body weight gain. This parameter was used to evaluate feed efficiency across different dietary treatments.

### Blood collection and analysis

At the end of the study, 3 mL of blood was collected from 3 birds per replicate the wing vein of both control and heat-stressed birds using sterile syringes (Kalsoom et al., 2024). Two separate tubes were prepared for analysis: one containing EDTA for differential leukocyte count (DLC) and one without EDTA for serum separation. The non-EDTA samples were centrifuged to obtain serum, which was analyzed for malondialdehyde (MDA) levels, while the EDTA-treated samples were used to assess DLC as indicators of oxidative stress and immune response.

### Serum malondialdehyde (MDA)

The concentration of MDA, a key marker of lipid peroxidation and oxidative stress, was measured using an MDA Assay Kit based on the thiobarbituric acid (TBA) reaction, following the method of Placer et al. (1966). In this assay, MDA reacts with TBA to form a stable pink chromophore, which was quantified spectrophotometrically at 532 nm.

### Differential Leukocyte Count (DLC)

Fresh blood smears were prepared by placing a drop of blood on a clean slide, spreading it evenly, and allowing it to air dry. Duplicate slides were fixed with methanol (2–3 minutes) and stained using a 2:3 Wright-Giemsa solution (15 minutes). The stained slides were examined under a light microscope (40X oil immersion lens, LAS X system) for leukocyte differentiation and quantification.

### Cecal Microbiota Evaluation

On day 42, two birds per replicate were randomly selected and humanely euthanized by cervical dislocation, following institutional ethical guidelines, to collect cecal contents for microbial analysis. Approximately 1 g of cecal digesta was homogenized in buffered peptone water (1:10 dilution) and cultured on selective agar media specific to each bacterial species. Lactobacillus spp. were incubated on MRS agar at 37°C with 5 % CO₂ for 24 hours. Bacterial colonies were counted using a colony counter, and results were expressed as log₁₀ colony-forming units (CFU/g).

### Statistical Analysis

The data were analyzed using a Completely Randomized Design (CRD). Results were expressed as mean ± standard error (SE). A one-way analysis of variance (ANOVA) was performed using SPSS software (version 21) to assess significant differences among treatment groups. Mean comparisons were conducted using Tukey’s Honestly Significant Difference (HSD) test at a significance level of *P* < 0.05, For microbiota counts, log-transformed values (log₁₀ CFU/g) were used before statistical analysis.

## Results

The feed intake (FI) of broilers subjected to heat stress is presented in Table 2. During the starter phase (days 1–21), no significant differences were observed among treatments (*P* > 0.05). However, in the finisher phase (days 22–42), FI increased significantly in birds supplemented with higher levels of Bacillus subtilis (4 × 10¹⁰ and 5 × 10¹⁰ CFU/kg) combined with 1 % garlic powder compared to the control (*P* < 0.001). This trend was reflected in cumulative FI for the finisher phase and the overall experimental period, indicating improved feed consumption with higher supplementation levels.

**Table 2:** Effect of probiotic (*Bacillus Subtilus*) and *Allium Sativum* (garlic) on feed intake (g/bird) during heat stress.

Groups	A (Control)	B (3 × 10^10^cfu/kg and 1 % garlic)	C (4 × 10^10^cfu/kg and 1 % garlic)	D (5 × 10^10^cfu/kg and 1 % garlic)	SEM	*P*-value
Week-1	125	127	127	126	0.53	0.51
Week-2	335	330	329	329	0.99	0.07
Week-3	543	538	537	535	1.31	0.27
Starter (d1-21)	1011	1003	1001	999	1.72	0.06
Week-4	849	850	853	862	2.02	0.09
Week-5	977^b^	983^b^	995^a^	999^a^	2.50	0.001
Week-6	1074^b^	1080^b^	1116^a^	1129^a^	5.61	0.001
Finisher (d21-42)	2900^b^	2913^b^	2964^a^	2990^a^	7.55	0.001
Overall(d1-42)	3911^b^	3916^b^	3965^a^	3989^a^	6.88	0.001

Heat stress was applied from day 22 to 42 by increasing the temperature to 32–35°C (average 34°C) for 3 hours daily. All treatment groups were exposed to heat stress, except the control group, which was maintained under thermoneutral conditions.

a Means having different superscript in the rows are significantly different (*P* < 0.05).

b Means having different superscript in the rows are significantly different (*P* < 0.05).

Similarly, weight gain (WG) did not differ significantly among groups during the starter phase (*P* > 0.05) but was markedly enhanced from week 4 onward in the higher supplementation groups (Table 3). Cumulative WG in the finisher phase and total WG (days 1–42) were significantly greater in birds receiving 4 × 10¹⁰ and 5 × 10¹⁰ CFU/kg B. subtilis + garlic compared to the control (*P* < 0.001).

**Table 3:** Effect of probiotic (*Bacillus subtilus*) and *Allium sativum* (garlic) on weight gain (g/bird) during heat stress.

Groups	A (Control)	B (3 × 10^10^cfu/kg and 1 % garlic)	C (4 × 10^10^cfu/kg and 1 % garlic)	D (5 × 10^10^cfu/kg and 1 % garlic)	SEM	*P*-value
Week-1	126	130	119	132	2.86	0.11
Week-2	294	292	291	291	0.70	0.89
Week-3	393	397	385	400	7.43	0.70
Starter (d 1-21)	770	776	795	780	7.21	0.101
Week-4	500^c^	506^b^^,^^c^	513^a^^b^	523^a^	4.94	<0.001
Week-5	595^c^	602^c^	614^b^	618^a^	3.52	<0.001
Week-6	656^c^	657^c^	681^b^	694^a^	5.31	<0.001
Finisher(d21-42)	1751^c^	1765c	1801^b^	1835^a^	8.69	<0.001
Overall(d1-42)	2521^c^	2541^c^	2604^b^	2615^a^	15.9	<0.001

Heat stress was applied from day 22 to 42 by increasing the temperature to 32–35°C (average 34°C) for 3 hours daily. All treatment groups were exposed to heat stress, except the control group, which was maintained under thermoneutral conditions.

a Means having different superscript in the rows are significantly different at α=0.05.

b Means having different superscript in the rows are significantly different at α=0.05.

c Means having different superscript in the rows are significantly different at α=0.05.

Feed conversion ratio (FCR) followed a similar pattern (Table 4). No differences were observed during the starter phase; however, during the finisher phase, Groups C and D (higher probiotic + garlic levels) exhibited significantly improved feed efficiency compared to the control (*P* < 0.001). Overall FCR for the 42-day trial was lowest in these groups, confirming their superior efficiency under heat stress conditions.

**Table 4:** Effect of probiotic (*Bacillus subtilus*) and *Allium sativum* (Garlic) on feed conversion ratio (FCR) during heat stress.

Groups	A (Control)	B (3 × 10^10^cfu/kg and 1 % garlic)	C (4 × 10^10^cfu/kg and 1 % garlic)	D (5 × 10^10^cfu/kg and 1 % garlic)	SE Mean	*P*-value
Week1	1.14	1.12	1.13	1.09	0.010	0.21
Week2	1.24	1.22	1.23	1.22	0.001	0.74
Week3	1.43	1.40	1.42	1.39	0.023	0.06
Starter (d 1-21)	1.31	1.30	1.29	1.28	0.015	0.10
Week4	1.72^a^	1.70^a^	1.67^b^	1.65^b^	0.017	<0.001
Week5	1.72^a^	1.71^a^	1.70^a^	1.66^b^	0.013	0.001
Week6	1.71^a^	1.71^a^	1.72^a^	1.64^b^	0.018	<0.001
Finish (d 21-42)	1.66^a^	1.65^a^^b^	1.63^c^	1.61^b^	0.015	<0.001
Overall (d 1-42)	1.558^a^	1.549^a^^b^	1.520^c^	1.49^b^	0.017	<0.001

Heat stress was applied from day 22 to 42 by increasing the temperature to 32–35°C (average 34°C) for 3 hours daily. All treatment groups were exposed to heat stress, except the control group, which was maintained under thermoneutral conditions.

a Means having different superscript in the rows are significantly different (*P* < 0.05).

b Means having different superscript in the rows are significantly different (*P* < 0.05).

Oxidative stress, as indicated by malondialdehyde (MDA) levels, was significantly reduced by supplementation (Table 5). The control group exhibited the highest MDA concentrations, while Groups C and D showed substantial reductions (*P* < 0.0001), suggesting enhanced antioxidant protection.

**Table 5:** Effect of probiotic (*Bacillus subtilus*) and *Allium sativum* (Garlic) on malondialdehyde (MDA; nmol/mg protein) and cecal microbial count (CFU log_10_/g) during heat stress.

Groups	MDA (nmol/mg protein)	*Escherichia coli*	*Salmonella*	*Lactobacillus spp*
Control (A)	12.17^a^±0.19	6.88 ± 0.03	6.82 ± 0.09	7.34 ± 0.04
B (3 × 10^10^cfu/kg and 1 % garlic)	8.64^b^±0.35	6.78 ± 0.01	6.88± 0.01	7.28± 0.03
C (4 × 10^10^cfu/kg and 1 % garlic)	7.64^b^^,^^c^ ± 0.06	6.85 ± 0.01	6.79± 0.03	7.32± 0.01
D (5 × 10^10^cfu/kg and 1 % garlic)	6.54^c^±0.34	6.80± 0.01	6.83± 0.01	7.39± 0.02
p value	0.001	0.11	0.91	0.77

Heat stress was applied from day 22 to 42 by increasing the temperature to 32–35°C (average 34°C) for 3 hours daily. All treatment groups were exposed to heat stress, except the control group, which was maintained under thermoneutral conditions.

a Means having different superscript in the rows are significantly different (*P* < 0.05).

b Means having different superscript in the rows are significantly different (*P* < 0.05).

c Means having different superscript in the rows are significantly different (*P* < 0.05).

Cecal microbiota analysis revealed no significant differences among groups for Escherichia coli, Salmonella, or Lactobacillus spp. counts (*P* > 0.05), although Lactobacillus levels were numerically higher in supplemented groups.

Differential leukocyte count (DLC) was significantly affected by dietary supplementation (Table 6). Lymphocyte percentages decreased with higher supplementation levels, while heterophil counts declined and monocyte and eosinophil counts increased in Groups C and D compared to the control (*P* < 0.05). Basophils were significantly reduced in supplemented groups. These hematological changes suggest improved immune modulation under heat stress conditions with probiotic and garlic supplementation.

**Table 6:** Effect of probiotic (*Bacillus subtilus*) and *Allium sativum* (Garlic) on Differential Leukocytes count (%) during environmentally induced heat stress.

	Lymphocytes (%)	Heterophils (%)	Monocytes (%)	Eosinophil (%)	Basophil (%)
A	62.75^a^±0.357	51.63^a^±0.842	2.30^c^±0.35	2.00^c^±0.00	4.34^a^±0.35
B	60.34^b^±0.201	49.03^b^ ±2.21	3.00^b^^,^^c^ ± 0.00	2.65^b^^,^^c^ ± 0.335	4.00^a^±0.01
C	59.75^c^±0.527	46.50^b^^,^^c^ ± 1.487	3.31^b^±0.31	3.00^b^±0.00	3.33^b^±0.01
D	53.25^d^±1.493	43.33^c^±0.35	4.57^a^±0.0.47	3.65^a^±0.335	2.66^c^±0.35
P-Value	0.021	0.012	0.013	0.020	0.034

Heat stress was applied from day 22 to 42 by increasing the temperature to 32–35°C (average 34°C) for 3 hours daily. All treatment groups were exposed to heat stress, except the control group, which was maintained under thermoneutral conditions.

a Means having different superscript in the rows are significantly different (*P* < 0.05).

b Means having different superscript in the rows are significantly different (*P* < 0.05).

c Means having different superscript in the rows are significantly different (*P* < 0.05).

## Discussion

Heat stress, when combined with high stocking density, poses a major challenge in poultry production by impairing growth performance and overall welfare (Abudabos et al., 2013). The present study demonstrated that dietary supplementation with *Bacillus subtilis* and garlic at higher inclusion levels (4 × 10¹⁰ and 5 × 10¹⁰ CFU/kg + 1 % garlic powder) significantly improved FI, WG, and FCR in heat-stressed broilers, particularly during the finisher phase. However, because garlic was included at a fixed level (1 %) across all probiotic treatments, it is difficult to determine the independent effect of garlic powder alone. This limitation should be considered in the interpretation of results.

The observed increase in FI during the finisher phase in *Bacillus subtilis* and garlic-supplemented groups aligns with previous findings that probiotics enhance gut microbiota balance, improve gut morphology, and increase nutrient digestibility, which may stimulate appetite (Usman et al., 2024). *Bacillus subtilis* has been reported to enhance intestinal villi height and crypt depth, facilitating greater nutrient absorption and compensating for heat stress-induced malabsorption (Mushtaq et al., 2025). Garlic supplementation, on the other hand, is reported to exert prebiotic effects, modulate gut microflora, and improve digestive enzyme activity, which may synergize with probiotics to enhance nutrient utilization under heat stress (Hafeez et al., 2023; Basit et al., 2025). Additionally, garlic contains organosulfur compounds, such as allicin and S-allyl cysteine, known to stimulate digestive enzyme secretion and modulate gut motility, further supporting improved FI under heat stress (Khan et al., 2012a). The significant increase in FI during weeks 5 and 6 in Groups C and D may be attributed to the gut microbial modulation by *Bacillus subtilis*, reducing harmful bacteria while promoting beneficial *Lactobacillus* spp., which enhances feed utilization (Mehmood et al., 2023). Furthermore, garlic's antioxidant properties may have alleviated oxidative stress-induced anorexia by reducing corticosterone levels, a known appetite suppressant under thermal stress (Khan et al., 2012a).

Broilers in Groups C and D exhibited significantly higher WG, particularly during the finisher phase, consistent with previous studies showing probiotics improve energy metabolism and protein synthesis, enhancing growth rates under environmental stressors (Ullah et al., 2023). The improved intestinal barrier function and reduced gut inflammation in probiotic-fed birds likely enhanced nutrient absorption, contributing to greater body weight gain (Dabool et al., 2024). Garlic supplementation further reinforced growth performance by acting as a natural growth promoter, as its bioactive compounds, including diallyl sulfides and flavonoids, are known to stimulate insulin-like growth factor-1 (IGF-1) expression, enhancing protein deposition and muscle growth (Ali et al., 2019). The improved gut microbiome balance from probiotic supplementation may have also contributed by reducing endotoxin production from pathogenic bacteria, thereby preventing growth depression typically observed under heat stress (Ahmad et al., 2022). The significant reduction in FCR observed in Groups C and D suggests enhanced feed efficiency, likely due to improved nutrient digestibility and intestinal health from probiotic and garlic supplementation. *Bacillus subtilis* has been shown to increase the secretion of digestive enzymes, including proteases and amylases, improving protein and starch digestibility, which directly impacts FCR (Mushtaq et al., 2025). The observed improvements in FCR during weeks 4–6 coincide with previous studies reporting that probiotics enhance intestinal microflora composition, reducing gut inflammation and energy loss due to immune activation, ultimately leading to better feed utilization (Mehmood et al., 2023; Ullah et al., 2023). The antioxidant properties of garlic may have further contributed by reducing oxidative stress-induced tissue damage, ensuring more efficient energy utilization for growth rather than stress adaptation (Khan et al., 2012a).

Heat stress induces oxidative damage in broilers by increasing reactive oxygen species (ROS) production, leading to lipid peroxidation and cellular dysfunction (Khan et al., 2023). The significant reduction in MDA levels in birds supplemented with *Bacillus subtilis* and garlic suggests an improved antioxidant defense mechanism under thermal stress conditions. These findings align with previous studies reporting that probiotics enhance enzymatic antioxidant activity (superoxide dismutase, glutathione peroxidase), thereby reducing lipid peroxidation (Dabool et al., 2024).

Garlic contains bioactive sulfur compounds such as allicin and S-allyl cysteine, which have been shown to scavenge ROS and inhibit lipid peroxidation, leading to reduced MDA concentrations in heat-stressed broilers (Abdelfattah et al., 2024). Additionally, *Bacillus subtilis* supplementation has been reported to increase gut barrier integrity, thereby reducing systemic inflammation and oxidative stress markers (Usman et al., 2024; Yu et al., 2024b). The significant decrease in MDA levels in Groups C and D suggests that higher probiotic and garlic supplementation enhances cellular resilience against oxidative damage, improving the birds’ overall physiological status during heat stress.

Despite the known modulatory effects of probiotics on gut microbiota, the present study found no significant differences in *Escherichia coli, Salmonella*, or *Lactobacillus* spp. counts among the treatment groups. This may be due to short-term supplementation or the absence of pathogenic microbial challenges, which could have influenced microbial dynamics more prominently. Although *Lactobacillus* spp. counts were numerically higher in probiotic-fed groups, the difference was not statistically significant. Previous studies have shown that *Bacillus*-based probiotics can promote the growth of beneficial bacteria while suppressing pathogenic enteric flora through competitive exclusion and antimicrobial peptide production (Dablool et al., 2024). Garlic’s prebiotic properties—which enhance short-chain fatty acid (SCFA) production—may have contributed to the minor increase in *Lactobacillus* spp., but longer feeding durations may be required to observe significant microbiota shifts (Khan et al., 2012a; Ali et al., 2019; Hafeez et al., 2024). It is also important to note that the current study employed culture-based enumeration methods, which, while practical, may lack the sensitivity to detect subtle shifts in microbial communities. Incorporating molecular techniques such as 16S rRNA sequencing in future studies would provide a more comprehensive characterization of gut microbial dynamics and the nuanced effects of dietary interventions.

Heat stress is known to cause immunosuppression by increasing circulating corticosterone levels, which leads to a shift in leukocyte populations, particularly an increase in heterophils and a decrease in lymphocytes (Khan et al., 2023). In the present study, lymphocyte percentages decreased with increasing probiotic and garlic supplementation, while heterophil counts significantly declined, indicating a reduction in heat stress-induced immune suppression. These findings are consistent with previous reports that probiotics help regulate stress-related immunomodulation by modulating cytokine expression and gut-associated lymphoid tissue (GALT) activity (Dabool et al., 2024).

Higher monocyte and eosinophil counts in the supplemented groups (C and D) suggest enhanced innate immune responses due to the immunostimulatory effects of *Bacillus subtilis* and garlic (Yu et al., 2024b). Monocytes play a crucial role in pathogen clearance and tissue repair, while eosinophils are involved in anti-inflammatory responses (Khan et al., 2012b). Garlic supplementation has been reported to enhance phagocytic activity, which may explain the increased monocyte counts in probiotic-fed groups (Khan et al., 2012a). The observed reduction in basophil counts in Groups C and D indicates lower systemic inflammation, as basophils are involved in allergic and stress-induced inflammatory reactions. This suggests that higher levels of *Bacillus subtilis* and garlic alleviated systemic stress responses, contributing to better health and performance in broilers exposed to heat stress.

## Conclusion

Supplementation with *Bacillus subtilis* and garlic significantly enhanced feed intake, weight gain, and feed conversion efficiency in heat-stressed broilers, with the group receiving 5 × 10¹⁰ CFU/kg *Bacillus subtilis* plus 1 % garlic demonstrating the best performance. This group also exhibited the lowest oxidative stress (MDA levels) and the most favorable immune profile, underscoring its potential as an effective nutritional strategy to alleviate heat stress in poultry production.

## Ethical statement

This study has been approved by the departmental committee on ethics and animal welfare the University of Agriculture, Peshawar (23/PS/2022).

## Consent to participate

All authors have given consent to participate this work.

## Consent for publication

All authors have given consent to publish this work.

## Availability of data and material

These data are available in student thesis.

## Funding

The author(s) reported there is no funding associated with the work featured in this article.

## AI information

The English of the paper was improved with the help of chatgpt which was read and revised.

## CRediT authorship contribution statement

**Hanan Al-Khalaifah:** Funding acquisition. **Muhammad Mushtaq:** Conceptualization. **Muhammad Shoib:** Methodology. **Imran Ullah:** Supervision. **Muqadar Shah:** Supervision. **Shabana Naz:** Software, Validation, Visualization. **Rifat Ullah Khan:** Writing – original draft, Writing – review & editing. **Ala Abudabos:** Formal analysis. **Ibrahim A. Alhidary:** Funding acquisition.

## Disclosures

Authors declare no conflict of interest
